# Evidence for a Specific Association Between Sustained Attention and Gait Speed in Middle-to-Older-Aged Adults

**DOI:** 10.3389/fnagi.2021.703434

**Published:** 2021-07-05

**Authors:** Hannah Park, Courtney Aul, Joseph DeGutis, On-Yee Lo, Victoria N. Poole, Regina McGlinchey, Jonathan F. Bean, Elizabeth Leritz, Michael Esterman

**Affiliations:** ^1^Boston Attention and Learning Laboratory (BALLAB), VA Boston Healthcare System, Boston, MA, United States; ^2^Department of Psychology, Brandeis University, Waltham, MA, United States; ^3^National Center for PTSD, VA Boston Healthcare System, Boston, MA, United States; ^4^Translational Research Center for TBI and Stress Disorders (TRACTS), VA Boston Healthcare System, Boston, MA, United States; ^5^Department of Psychiatry, Harvard Medical School, Boston, MA, United States; ^6^Hinda and Arthur Marcus Institute for Aging Research, Hebrew SeniorLife, Boston, MA, United States; ^7^Division of Gerontology, Beth Israel Deaconess Medical Center, Boston, MA, United States; ^8^Department of Medicine, Harvard Medical School, Boston, MA, United States; ^9^Rush Alzheimer's Disease Center and Department of Orthopedic Surgery, Rush University Medical Center, Chicago, IL, United States; ^10^New England Geriatric Research, Educational and Clinical Center (NEGRECC), VA Boston Healthcare System, Boston, MA, United States; ^11^Department of Physical Medicine and Rehabilitation, Harvard Medical School, Boston, MA, United States; ^12^Spaulding Rehabilitation Hospital, Boston, MA, United States; ^13^Department of Psychiatry, Boston University School of Medicine, Boston, MA, United States

**Keywords:** executive function, cognition, cognitive aging, sustained attention, gait speed

## Abstract

Although cognitive decline has previously been associated with mobility limitations and frailty, the relationship between sustained attention and gait speed is incompletely characterized. To better quantify the specificity of the sustained attention and gait speed association, we examined the extent to which this relationship is unique rather than accounted for by executive functioning and physical health characteristics. 58 middle-to-older-aged community-dwelling adults without overt evidence of cognitive impairment (45–90 years old; 21 females) participated in the study. Each participant completed a 4-meter gait speed assessment and validated neuropsychological tests to examine various domains of executive functioning including working memory (i.e., Digit Span), inhibitory control (i.e., D-KEFS Color-Word Interference), and task switching (i.e., D-KEFS Number/Letter Switching). Multiple physical and vascular risk factors were also evaluated. Sustained attention was assessed using the gradual onset continuous performance task (gradCPT), a well-validated go/no-go sustained attention task. A series of linear regression models were used to examine how different aspects of cognition, including sustained attention and traditional measures of executive functioning, related to gait speed while controlling for a variety of physical and vascular risk factors. Among all predictors, gradCPT accuracy explained the most variance in gait speed (*R*^2^ = 0.19, *p* < 0.001) and was the only significant predictor (β = 0.35, *p* = 0.01) when accounting for executive functioning and other physical and vascular risk factors. The present results indicate that sustained attention may be uniquely sensitive and mechanistically linked to mobility limitations in middle-to-older adults.

## Introduction

Across the older adult population mobility has been identified as a critical determinant of well-being. In particular, walking speed is an easy to execute, reliable, and valid indicator of functional capacity (Middleton et al., 2015) as well as overall health (Cesari et al., 2005; Studenski et al., 2011). Slow gait speed is one of Fried's defining criteria for the frailty phenotype (Fried et al., 2001), as it increases susceptibility to negative outcomes, including the development of physical disability (Clegg et al., 2013), and is a verified predictor of falls, morbidity, and mortality (e.g., Fried et al., 2001; Studenski et al., 2011; Welch et al., 2020). Diminished gait speed is further linked to increased risk for adverse cardiovascular events, including coronary heart disease (Dumurgier et al., 2009), and poor cognitive aging outcomes, such as dementia (Alfaro-Acha et al., 2007). Given its relationship to healthy aging, gait is considered the “sixth vital sign” (Fritz and Lusardi, 2009; Middleton et al., 2015) to assess functional status. As gait speed is associated with various factors that critically impact quality of life, understanding these multifaceted relationships has significant translational implications for older adults. The present study aims to better understand how specific aspects of cognition relate to gait speed.

Executive functions, including working memory, task switching, and inhibitory control (Miyake et al., 2000), are critical for fundamental aspects of daily life. Executive functioning deficits are often accompanied with functional disability (e.g., Hajjar et al., 2009), and there is an association between age-related mobility loss and executive decline (Hajjar et al., 2009; Vazzana et al., 2010; McGough et al., 2011). For instance, reduced task switching abilities have been directly related to diminished gait speed (Hirota et al., 2010; McGough et al., 2011) and future mobility impairment (Vazzana et al., 2010). These processes depend on several large-scale brain networks, especially frontal-parietal control networks, which are susceptible to age-related alterations (Campbell et al., 2012). Additionally, gait speed in older adults is associated with activity in executive control and attention networks (Jordan et al., 2017; Zhou et al., 2020), suggesting overlap in the neural correlates of these processes. As we age, walking may become less automatic due to multiple factors, including attenuated somatosensory input (Clark et al., 2014), which increases reliance on alternative resources to maintain optimal functioning. Executive functions may compensate for this reduction in automaticity, and therefore mediate the rate of decline (Yogev-Seligmann et al., 2008; Clark et al., 2014). However, if executive function capacity degrades due to general age-related or neurodegenerative processes, mobility loss will likely be exacerbated (Yogev-Seligmann et al., 2008). As executive dysfunction is a predictor of mobility impairment and is associated with negative outcomes in older adults, the interplay between executive and motor function has important implications for quality of life.

Previous work regarding the relationship between executive function and gait predominantly examines transient acts of cognitive control. Another critical aspect of cognition, less often characterized as an executive function, is the ability to *sustain* attentional control. The capacity to maintain task-set and goal-directed attention over time, or sustain attention, is necessary for various higher-level cognitive processes (Fortenbaugh et al., 2017b), including other aspects of executive functioning such as working memory and inhibitory control (deBettencourt et al., 2019). Sustained attention is also critical for everyday activities, such as safe driving (Yanko and Spalek, 2013), and is related to numerous functional outcomes, including motor function recovery following stroke (Robertson et al., 1997). The ability to sustain attention declines with age (Fortenbaugh et al., 2015) and the presence of cardiovascular risk factors (Wooten et al., 2019). Prior research demonstrates that sustained attention deficits are related to mobility loss, even in the absence of explicit cognitive impairment (O'Halloran et al., 2011, 2014; Killane et al., 2014). Studies examining gait and cognition found that poorer sustained attention, quantified by reaction time variability on the Sustained Attention to Response Task (SART), retrospectively predicted falls within the last year (O'Halloran et al., 2011), progression into frailty (O'Halloran et al., 2014), and reduced gait speed when the task required additional motor coordination (Killane et al., 2014). These findings are in line with the reduction of automaticity model, wherein cognitive resources moderate age-related mobility loss.

Expanding our comprehension of the relationship between sustained attention and gait is critical for understanding the aging process and for the development of interventions to attenuate functional decline. As such, there are several limitations of previous work relating sustained attention to mobility. First, while previous studies have demonstrated the importance of sustained attention in healthy aging, they have also revealed a relationship between reduced processing speed and other aspects of executive dysfunction with diminished gait speed (e.g., Killane et al., 2014). Thus, it is unclear if sustained attention is a unique predictor of gait that surpasses more global cognitive decline. Second, previous measures of sustained attention were based solely on reaction time variability (Killane et al., 2014; O'Halloran et al., 2014). Although reaction time variability is a marker of sustained attention (Fortenbaugh et al., 2015), it may also be related to impaired motor processes or processing speed, therefore task accuracy is potentially a more direct sustained attention measure. Additionally, previous studies have relied on a single, albeit well-characterized, measure of sustained attention, the SART (O'Halloran et al., 2011, 2014; Killane et al., 2014), and convergence using alternative measures would provide more compelling evidence for a robust relationship between sustained attention and mobility. While the SART is a well-established assessment of sustained attention, we have developed the gradual-onset continuous performance task (gradCPT) (Esterman et al., 2013), which eliminates the exogenous effects of abrupt onsets and isolates endogenous attentional control (Fortenbaugh et al., 2017b). This task is a well-validated and sensitive measure of sustained attention, with established lifespan trajectories (Fortenbaugh et al., 2015) and sensitivity to a number of clinical disorders (e.g., Esterman et al., 2019). Finally, it is unclear whether the relationship between sustained attention and mobility is unique when controlling for health factors such as a comprehensive cardiovascular risk profile, which is also associated with sustained attention impairments (Wooten et al., 2019), and mobility loss (Hajjar et al., 2009). Thus, we aimed to replicate and extend previous work linking sustained attention and gait using gradCPT accuracy (*d*′) as our primary measure. We then investigated whether the predictive power of sustained attention was greater than traditional neuropsychological measures of executive functioning, while controlling for a range of demographic and health factors. We hypothesized that sustained attention would uniquely predict gait speed.

## Methods

### Study Design and Participants

Sixty-eight participants from the Cerebrovascular Integrity and Risk for Cognitive decline in Aging (CIRCA) study completed the assessments required for the present study. CIRCA was a cross-sectional study, conducted between 2014 and 2018, investigating the effects of vascular risk factors on cognition in middle-to-older-aged adults (Wooten et al., 2019). Recruitment targeted those at high risk for metabolic syndrome (MetS) directly through the Department of Veterans Affairs Healthcare System and through advertisement in the greater Boston, MA metropolitan area. Eligibility requirements consisted of the ability to communicate in English and being between the ages of 45–90. Individuals were excluded if they presented with cognitive impairment (MMSE score ≤ 24), reported the presence of a significant medical disease, prior major surgery, head trauma, neurological disorders, a history of severe or current psychiatric disorders, a history or current diagnosis of drug abuse or dependence, or contraindication to MRI. Although collected, MRI scans were not analyzed in the present study. Study visits were completed over 1 or 2 days, depending on participants personal choice and schedules. Cognitive testing was administered in a quiet room by a research assistant trained by a board-certified neuropsychologist. The study protocol was approved by the Institutional Review Board of the Department of Veterans Affairs (VA) Boston Healthcare System, and all participants provided informed consent before study procedures.

### Physical Performance

The Short Physical Performance Battery (SPPB) contains three functional tasks to examine balance, gait, and chair stand abilities (Guralnik et al., 1994). As performance of many SPPB tasks were at ceiling for most subjects, the current study focused exclusively on walking score, which was based on walking time over a 4-meter course. Participants were instructed to walk at their usual pace at the examiner's command, as if walking down the street to go to the store, and they completed this assessment twice. The average time from the two trials was used for all analyses. Gait speed, our primary mobility measure, was calculated by dividing the distance by walking time.

### Gradual-Onset Continuous Performance Task

The gradCPT is a well-validated measure of sustained attention that has been used to investigate individual differences and lifespan trajectories in thousands of web-based participants (e.g., Fortenbaugh et al., 2015), neuropsychiatric populations (e.g., Fortenbaugh et al., 2017a; Esterman et al., 2019), and lab-based studies of aging (Wooten et al., 2019). During this validated 4-min version of the task (Fortenbaugh et al., 2015), participants were shown a series of gray-scale scene images that gradually transitioned approximately every 800 ms using linear pixel-by-pixel interpolation. Participants respond via button press to frequently occurring city images (90% of stimuli) and withhold responses to rare mountain images (10% of stimuli). Each participant had three 30 s practice sessions before the task.

Accuracy (*d'*) is the primary measure of sustained attention on the gradCPT (Fortenbaugh et al., 2015), and therefore was considered the primary measure in the current study. *d*' is calculated using signal detection theory and is based on false alarm rate (omission errors, or failure to respond to cities) as well as miss rate (commission errors, or failure to withhold responses to mountains). Raw accuracy (omission and commission errors) is also reported. Mean reaction time (RT; in milliseconds) of the correct responses to cities was included in the subsequent analyses to control for general speed of motor responses, overall strategy, and possible speed-accuracy trade-offs (Fortenbaugh et al., 2015). Data exclusion criteria were the same as our previously established guidelines (Fortenbaugh et al., 2015; Esterman et al., 2019; Wooten et al., 2019), such that data from participants who did not respond for a period of 30 s or more were discarded. Nine participants were excluded based on this criterion.

### Neuropsychological Assessment

Participants completed a comprehensive neuropsychological battery to assess broad cognitive domains. The following measures assessed attention, memory, and executive function: D-KEFS Number/Letter Switching (Delis et al., 2001), Digit Span subtest from the Wechsler Adult Intelligence Scale (Wechsler, 1997), Controlled Oral Word Association (Spreen and Strauss, 1998), the Logical Memory subtest from the Wechsler Memory Scale-Third Edition (Wechsler, 1997), the California Verbal Learning Test-Second Edition (Delis et al., 2001), the Boston Naming Test (Kaplan et al., 1983) and the D-KEFS Color-Word Interference Test (Delis et al., 2001). We chose Digit Span Sequencing (Wechsler, 1997), D-KEFS Color-Word Inhibition (Delis et al., 2001), and D-KEFS Number/Letter Switching (Delis et al., 2001) to reflect the executive function subcomponents of working memory, inhibitory control, and task switching, respectively (Miyake et al., 2000). We used scaled scores to account for participant's age. To further test the specificity of cognitive relationships with gait, premorbid IQ was estimated from the Wechsler Test of Adult Reading (WTAR) (Wechsler, 2001).

### Health and Physical Factors

Participants were evaluated for general health/physical status, including the six MetS risk factors: waist circumference (cm), triglycerides (mg/dL), high density lipoprotein-cholesterol (mg/dL), systolic and diastolic blood pressure (mmHg), and fasting plasma glucose (mg/dL). For all MetS risk factors, the raw values were used as continuous. One participant was excluded based on a triglyceride level > 7 SD from the mean. Height (cm) was also assessed as a factor with a potential impact on gait speed.

### Statistical Analysis

We evaluated three sets of models to predict gait speed. First, we used linear regression to assess whether gait speed was associated with gradCPT performance (*d*′). To account for the effect of reaction time (RT) on task accuracy and walking speed, mean task RT was included as a covariate in the same model. Second, we repeated the gait analyses using the individual executive functioning measures: working memory, inhibitory control, and task switching. Third, we included significant neuropsychological measures in the gradCPT model to determine if gradCPT could explain gait speed above and beyond these measures of executive functioning. In all models, we considered several possible demographic and physical factors as covariates, including age, sex, years of education, premorbid IQ, and health factors (i.e., height, waist circumference, triglycerides, cholesterol, systolic and diastolic blood pressure, and glucose levels). We included these factors in the models if they significantly correlated with gait speed via Pearson correlations (*p* < 0.05). Prior to these analyses, variables with high skewness (> |1|) were transformed to produce a more normal distribution (skewness < |1|), using the method that best corrected for the skew (log, square root, and inverse tangent). The WTAR scaled score and triglyceride estimates were log transformed. Glucose underwent an inverse tangent transformation, and omission errors and commission errors were square root transformed. Transformed variables were used in all analyses except for the descriptive statistics. Finally, as an alternative to the above *a priori* model selection, we conducted a stepwise regression (with MATLAB *stepwiselm*, which uses forward and backward stepwise regression to determine a final model) that selected amongst all factors to predict gait speed.

## Results

### Demographics and Correlations

**Figure 1:**
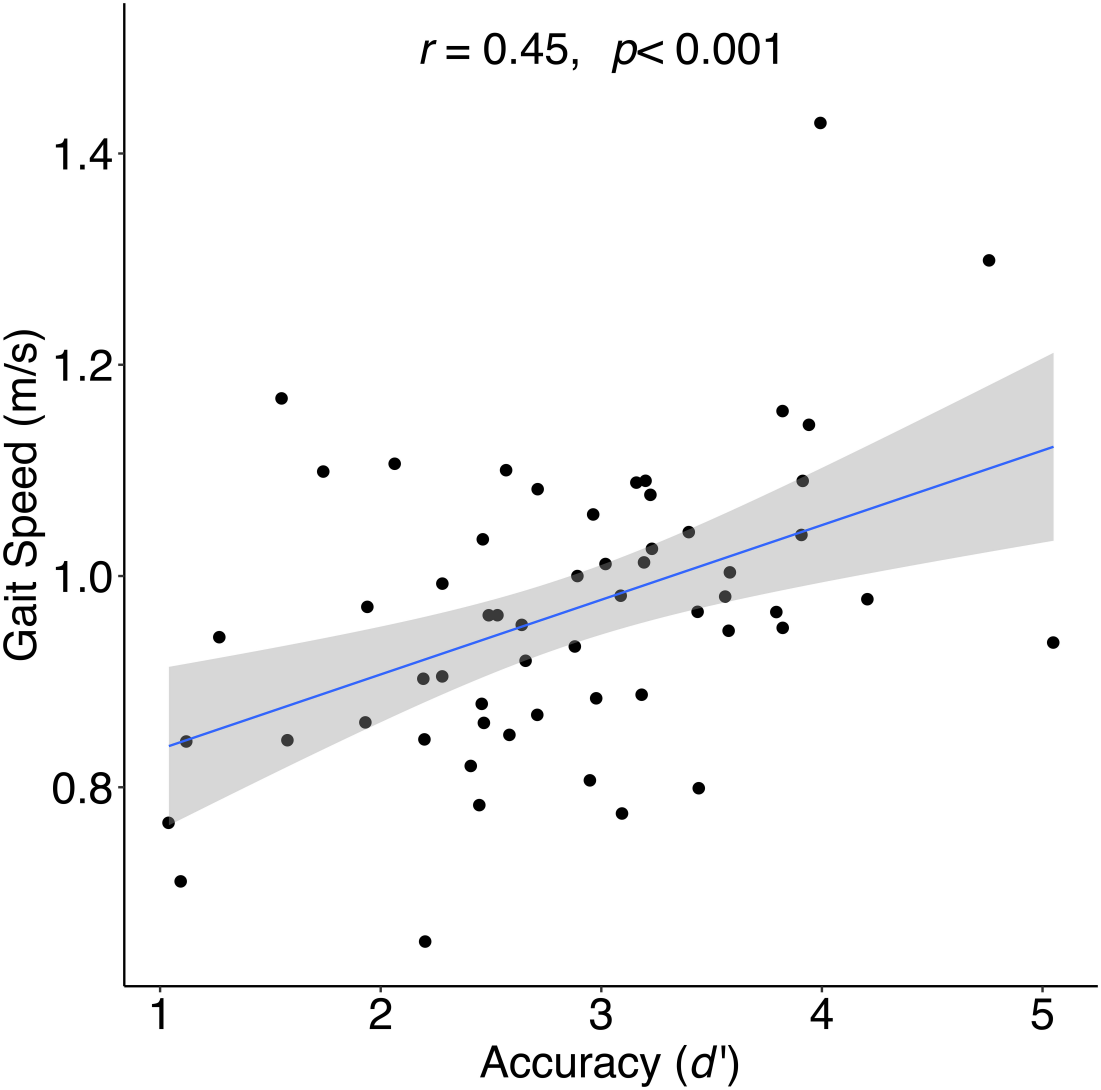
Correlation between sustained attention (gradCPT accuracy [*d*′]) and gait speed for during 4-meter walk at preferred walking speed.

Of the 68 initial participants, nine were excluded for non-compliance on the gradCPT, and one for an extreme outlier in health factors (see Methods). The following analyses were therefore conducted with 58 participants. The average age for the remaining participants was 62.16 years (*SD* = 9.89), 21 were female, and the average duration of education was 16.47 years (*SD* = 2.59). The average gait speed was 0.97 m/s (*SD* = 0.14). Table 1 contains participant characteristics and correlations between gait speed and all variables (skewed variables were transformed; see Methods). We observed significant correlations between gait speed and gradCPT accuracy (*d'*; *r* = 0.45, *p* < 0.001; Figure 1), mean RT (*r* = −0.31, *p* = 0.02), omission error rate (*r* = −0.31, *p* = 0.02), commission error rate (*r* = −0.30, *p* = 0.02), Number Letter/Switching scaled score (*r* = 0.32, *p* = 0.01), education (*r* = 0.32, *p* = 0.02), and waist circumference (*r* = −0.29, *p* = 0.03). As expected, *d'* correlated highly with commission (*r* = −0.67, *p* < 0.0001) and omission (*r* = −0.75, *p* < 0.0001) errors. Bonferroni adjusted *p*-values were calculated to correct for multiple comparisons (*p* < 0.0028). Only *d'* survived this correction (*p* < 0.001), however, our hypotheses regarding the potential effects of demographic and health factors led us to include zero order correlations with uncorrected *p*-values <0.05 as covariates. Thus, waist circumference and education were included as covariates in all subsequent analyses to isolate the relationship between gait and cognitive functioning.

**Table 1:** Sample characteristics and correlations with gait speed.

**Sample size**	***Mean***	***SD***	**Pearson's *r***
	***N*** **=** **58**		***N* = 58**
Age (years)	62.16	9.89	0.01
Male (%)	63.79		*t*_57_ = 0.58^1^
Education (years)	16.47	2.59	0.32^*^
WTAR Estimated FSIQ^a^	115.62	14.56	−0.24
D-KEFS number/letter switching scaled score	10.05	3.57	0.32^*^
Digit span sequencing scaled score	11.19	2.67	0.17
D-KEFS color-word inhibition scaled score	10.71	3.17	0.24
Height (cm)	67.35	3.75	−0.04
Waist circumference (cm)	96.37	17.26	−0.29^*^
Triglyceride^a^ (mg/dL)	97.86	42.92	0.21
HDL-cholesterol (mg/dL)	58.29	16.91	0.15
Systolic blood pressure (mmHg)	126.28	16.95	−0.21
Diastolic blood pressure (mmHg)	73.69	9.41	−0.02
Glucose^a^ (mg/dL)	95.76	15.67	0.08
Gait Speed (meters/second)	0.97	0.14	—
gradCPT accuracy (*d′*)	2.84	0.87	0.45^***^
gradCPT mean reaction time (RT)	0.82	0.07	−0.31^*^
gradCPT omission Errors^a^	0.06	0.07	−0.31^*^
gradCPT commission Errors^a^	0.20	0.16	−0.30^*^

* *p < 0.05*,

** *p < 0.01*,

*** *p < 0.001*.

a *Transformed due to skewness (see Methods). ^1^T-Test on gait speed by sex*.

### Sustained Attention and Gait Speed

The overall regression model predicting gait speed from sustained attention (*d'*) with covariates waist circumference, education, and gradCPT mean RT was significant (adjusted *R*^2^ = 0.24, *p* < 0.001), and the only significant predictor was *d'* (β = 0.30, *p* = 0.03; Table 2). These results demonstrate a linear relationship between gait speed and *d'*, that survives after accounting for the effect of overall task speed (mean RT), along with demographic, health, and physical factors.

**Table 2:** Linear regressions predicting gait speed.

**Domain**	**Adjusted *R*^**2**^**	**Predictors**	**ß**	***p*-value**
Sustained Attention	0.24^***^	gradCPT *d′*	0.30	0.03^*^
		RT	−0.20	0.11
		Waist circumference	−0.16	0.22
		Education	0.16	0.21
Executive Functioning	0.12^*^	Task switching^†^	0.23	0.12
		Waist circumference	−0.21	0.13
		Education	0.11	0.50
	0.09	Working memory^††^	0.05	0.71
		Waist circumference	−0.19	0.18
		Education	0.23	0.12
	0.12^*^	Inhibitory control^†††^	0.19	0.14
		Waist circumference	−0.20	0.14
		Education	0.20	0.12
Sustained Attention & Executive Functioning	0.21^**^	gradCPT *d'*	0.35	0.01^**^
		Task switching^†^	0.08	0.62
		Waist circumference	−0.15	0.25
		Education	0.11	0.46
Stepwise Model^1^	0.19^***^	gradCPT *d′*	0.45	0.0004^***^

* *p < 0.05*,

** *p < 0.01*,

*** *p < 0.001*.

† *D-KEFS Number/Letter Switching (scaled score)*,

†† *Digit Span Sequencing (scaled score)*,

††† *D-KEFS Color-Word Inhibition (scaled score)*.

1 *Via stepwise regression analysis (see Methods)*.

### Executive Function and Gait Speed

The overall regression models for all executive function measures were significant (task switching: adjusted *R*^2^ = 0.12, *p* = 0.02; working memory: adjusted *R*^2^ = 0.09, *p* = 0.05; inhibitory control: adjusted *R*^2^ = 0.12, *p* = 0.02). Despite model significance, task switching (Number/Letter Switching), working memory (Digit Span), and inhibitory control (Color-Word Inhibition) were not significant predictors of gait speed, indicating that gait speed is not significantly associated with these domains when considering demographic, health, and physical factors. Notably, task switching showed a zero-order correlation with gait speed, consistent with previous studies.

### Combined and Stepwise Models

To further explore the relationship between gait and sustained attention, we repeated the gradCPT regression and included task switching (Number/Letter Switching). This model was used to determine if sustained attention predicted gait speed when accounting for executive functioning, education, and waist circumference. We included task switching, even though it was not a significant predictor in the regression model (see above; Table 2), as it was significantly correlated with gait speed independently (Table 1). This overall regression model was significant (adjusted *R*^2^ = 0.21, *p* = 0.003), and there was a main effect of *d'* (β = 0.35, *p* = 0.01; Table 2). Finally, to determine if there was a simpler model, without a priori predictors, that explained gait speed, based on all factors, we conducted a stepwise regression analysis (see Methods). The selected model included only *d'* (adjusted *R*^2^ = 0.19, *p* = 0.0004), suggesting that the relationship between sustained attention and gait speed is independent of executive functioning, physical, and health factors.

## Discussion

The present study demonstrates a relationship between sustained attention and gait speed that extends beyond traditional neuropsychological measures of executive functioning. Those who achieved higher accuracy (*d*') on the gradCPT walked faster, even when accounting for several demographic, physical, and health-related factors. This same relationship was not robust when examining other aspects of executive function (i.e., task switching, working memory, inhibitory control) suggesting greater specificity for the relationship between sustained attention and mobility. Our findings replicate and extend previous work (Killane et al., 2014), clarifying the association between sustained attention and gait speed by controlling for executive functioning and vascular risk factors. Given the relationship between gait and functional outcomes in older adults, further investigation of sustained attention as a potential mediator of mobility decline is warranted.

As there are several distinct aspects of executive functioning, it follows that they do not equally compensate for mobility loss. Our results suggest sustained attention relates to mobility differently than other executive functions, as only sustained attention predicted walking speed when controlling for potential health and physical confounds. This specificity has critical implications as numerous negative functional outcomes are related to distinct, rather than global cognitive deficits (e.g., Robertson et al., 1997; Hirota et al., 2010; Vazzana et al., 2010; Huntley et al., 2017). As such, it is possible progression toward mobility loss may be uniquely delayed in individuals with preserved sustained attention, demonstrating its potential unique importance for functional capacity in older adults.

Sustained attention lapses are common during monotonous tasks and can be attributed to internal distractions, like mind wandering, as well as external distractions, and reduced arousal (Esterman and Rothlein, 2019). In older adults, however, incidences of mind wandering are not as frequent (Moran et al., 2021), suggesting that lapses are more appropriately ascribed to other factors, such as top-down control failures (Clapp and Gazzaley, 2012). Additionally, the observed accuracy (*d'*) effect was driven by both omission and commission errors (Table 1; Methods), which may indicate different types of lapses. Commission errors may reflect inhibitory control failures or mindless responding, while omission errors may reflect more catastrophic task disengagement or inefficient sensory processing, suggesting both may contribute to slowing of gait. As sensory functions decline, greater top-down control may be needed to enhance the noisy sensory representations required for the maintenance of mobility (Clark et al., 2014). The ability to continuously attend to task-relevant stimuli, such as visual and sensorimotor information, may be why sustaining attention is critical for walking. Sustaining attention and other aspects of top-down control rely on the dorsal attention network (DAN) along with its distinctiveness from the default mode network (DMN) (Fortenbaugh et al., 2018; Esterman and Rothlein, 2019). Stronger negative connectivity between task-positive (DAN) and task-negative (DMN) networks is associated with better sustained attention (Fortenbaugh et al., 2018; Rothlein et al., 2018), and has also been linked to mobility (Lo et al., 2017), suggesting reliance on partially overlapping networks. Thus, distilling the various factors that lead to sustained attention failures in older adults may help elucidate the sustained attention and gait association.

Reticular activating system (RAS) dysfunction may contribute to sustained attention failures (e.g., Unsworth and Robison, 2016; French and Muthusamy, 2018; Esterman and Rothlein, 2019) and gait impairments (e.g., French and Muthusamy, 2018). For instance, locus coeruleus dysfunction can cause attentional lapses through hypoarousal and reduced engagement, as well as hyperarousal and excessive distractibility (Unsworth and Robison, 2016; Esterman and Rothlein, 2019). Alternatively, pedunculopontine nucleus degeneration is associated with diseases where gait impairments are common, as well as attention and arousal impairments (French and Muthusamy, 2018). As both sustained attention and gait are independently linked to aspects of the RAS, this system could serve as an underlying mechanism for the observed gait and sustained attention relationship.

As sustained attention declines with age (Fortenbaugh et al., 2015; Wooten et al., 2019) and is associated with a range of functional outcomes, it follows that attention training programs could be introduced to preserve quality of life. Previous work demonstrates that sustained attention is responsive to training in older adult populations. For instance, a computer-based training program designed to improve sustained attention has been effective in healthy older adults (van Vleet et al., 2016). Additionally, a game-based neurofeedback training program improved sustained attention in healthy older adults and in those with amnesic mild cognitive impairment (Jirayucharoensak et al., 2019). Taken together with the present findings, it appears feasible to train sustained attention, and future work should determine if these cognitive training programs could delay the onset of mobility decline in healthy middle-to-older-aged adults.

We observed an additional relationship between task switching and gait speed, however it was not robust to accounting for waist circumference and education. Given that an association was present, as well as the previous literature, task switching likely also plays a role in mediating mobility loss. Alternatively, it is possible that subtle task switching deficits are related to decreases in walking speed, but standard neuropsychological measures of task switching are not sensitive enough to detect these subclinical impairments. Due to the relatively small sample size (*N* = 58), it is possible that the present study was underpowered, resulting in a failure to detect the association between task switching and gait speed. It is possible that if gait was assessed using a dual-tasking paradigm, or task switching was examined with more sensitive measures, then the relationship would be more robust.

The gradCPT relies on various functional systems beyond sustained attention, raising the concern that the relationship between gait speed and performance is related to other functions rather than sustained attention. For instance, to perform the task participants are required to make repeated button presses, which depends on motor control, and could drive the observed relationship between gait speed and sustained attention. However, the fact that accuracy predicted gait speed when controlling for mean reaction time suggests this motor component does not fully account for the association. Moreover, the gradCPT is uniquely visually demanding, requiring detection of subtle transitions between images. As the integrity of mid and low-level visual processes degrade with age (e.g., decreased contrast sensitivity; Owsley et al., 1983), visual functioning could also factor into the present results. While traditional neuropsychological tests require intact visual functioning, it is possible that the gradCPT is more sensitive to impairments, due to higher information processing speed demands. Although these visual-motor factors should be explored in future studies, the present results highlight the predictive power of visual sustained attention for mobility.

The present study has several limitations. First, the current sample is relatively small (*N* = 58), so replication in a larger population would be beneficial to examine these subclinical individual differences. Second, the neuropsychological tests used to examine task switching, working memory, and inhibitory control are not necessarily comparable to the gradCPT, and more sensitive cognitive tests should be used to examine these other aspects of executive function. Moreover, the current sample is relatively healthy, potentially limiting generalizability to a more clinically impaired group. Finally, causality cannot be determined with the present design; a longitudinal study is needed to conclude whether mobility critically depends on sustained attention.

Despite these limitations, the present work demonstrates a significant relationship between sustained attention and gait speed in those without explicit cognitive impairment. Individual differences in sustained attention appear to be reflected by variations in gait speed, suggesting that sustained attention may play a critical role in mobility decline. The gradCPT appears to be sensitive to subtle gait impairments, which suggests that it could be used to identify those at risk for future decline. The present study extends our understanding of the cognitive contributions to mobility and provides insight into targets for interventions to mediate decline.

## Data Availability Statement

The datasets presented in this article are not readily available because these data are owned by the United States Department of Veterans Affairs. The Department of Veterans Affairs will make this data publicly available and requests for the data can be made by interested individuals by filing a Freedom of Information Act request. Requests to access the datasets should be directed to The Privacy Officer at VA Boston Healthcare System, vhabhsFOIAofficers@va.gov or the FOIA Intake Center (see https://www.va.gov/FOIA/Requests.asp for more details).

## Ethics Statement

The studies involving human participants were reviewed and approved by Institutional Review Board of the Department of Veterans Affairs (VA) Boston Healthcare System. The patients/participants provided their written informed consent to participate in this study.

## Author Contributions

EL designed the study and obtained funding. EL and JB collected the data. HP and CA contributed equally to the statistical analyses and writing of the manuscript. JD, O-YL, RM, and VP critically revised and contributed to the manuscript content. ME oversaw all analyses, interpretation, and preparation of the manuscript. All authors contributed to and approved the final version.

## Conflict of Interest

The authors declare that the research was conducted in the absence of any commercial or financial relationships that could be construed as a potential conflict of interest.
